# Fostering Enterprise Performance Through Employee Brand Engagement and Knowledge Sharing Culture: Mediating Role of Innovative Capability

**DOI:** 10.3389/fpsyg.2022.921237

**Published:** 2022-07-13

**Authors:** Yaowen Zhang

**Affiliations:** School of Economics, Xi’an University of Finance and Economics, Xi’an, China

**Keywords:** enterprise performance, knowledge sharing culture, innovation, innovative capabilities, employee brand equity

## Abstract

Enterprise performance is a critical component of any organizational success that is directly affected by its employees and the culture prevailing in the organizations. In order to gain strategic advantage from the employee brand equity it is important that organizations make efforts in retaining such employees that benefit the organizations. Therefore, this research examines the impact of employee brand equity and knowledge sharing culture on the enterprise performance with the mediating role of innovative capabilities. A self-administered survey was conducted among the 323 employees of information technology sector working in the software houses in China. Smart PLS has been used to analyze the data through partial least square structural equation modeling. Results of the study have demonstrated that knowledge sharing culture plays a significant role in the enterprise performance while employee brand equity could not find statistically significant impact on enterprise performance. In addition, the SEM analysis further showed that employee brand equity and knowledge sharing culture play a significant role in the innovative capabilities. Results also revealed that innovative capabilities mediate the effect of employee brand equity and knowledge sharing culture variables on the enterprise performance. This research enriches the literature by examining the role of knowledge sharing culture in enterprise performance and innovative capabilities. This research further offers certain implications for the human resource department in developing their human resources. This can be achieved by availing the maximum skills of the branded employees by creating learning opportunities for the other employees through training sessions where they help and share their experiences.

## Introduction

Employee engagement is recognized as a persistent, positive, and pervasive work-related psychological phenomenon related to the dedication and vigor efforts taken by the employees toward the organization (Sharma et al., 2021). This type of proactive employee behavior is not only restricted to organizations but goes beyond job needs (Van Nguyen et al., 2021). In the present era, organizations operate in a highly competitive business environment. Due to thisreason, the firms require proactive employees for adapting, responding, and quickly engaging to the fierce market challenges (Paulsen, 2016). Employees’ willingness to engage in pro-active work engagement helps in overcoming the competitive challenges. The enterprise performance is highly dependent on the employees and their ability to serve the organization both effectively and efficiently. Additionally, the transfer of knowledge within the organization equally contributes to the success of the organization (Venkatesh et al., 2022). Research evidence reports that employee-related and organizational-related factors are essential for the growth and survival of modern business organizations in this highly competitive business environment.

Enterprise performance is a concept of public management practice and research that mainly deals with aspects band factors that boost the performance (Le and Le, 2019). However, this concept has been a debatable topic for a long time (Al Mamun and Fazal, 2018). This debate centered on the idea that enterprise performance is fostered by the effectiveness and efficiency of business operations. Enterprise performance is fostered by various factors that prevail in the organization. Researchers argued that every organization is finding effective ways to enhance enterprise performance because high performance leads toward completive advantage (Mutenyo et al., 2021). The management of the organizations devises different novel strategies in order to increase enterprise performance (Wang et al., 2022). According to Si and Si (2019), the role of employees is highly significant in boosting the performance of the organization. In the recent era, the role of innovation also plays a role in affecting enterprise performance (Li et al., 2019). The firms that invest in innovative strategies and innovation immensely boost the performance of the firm. Innovation within a firm should be highly encouraged by the management because innovation can speed up the business operations, hence increasing the level of efficiency. Pinheiro et al. (2020) asserted that the flow of information is necessary for bringing innovation to the organization and maximizing enterprise performance.

Employee brand engagement is activity related to the employees’ involvement in the organization’s brand (Van Nguyen et al., 2021). This engagement shows that employees are willing to improve the overall performance of the enterprise by achieving the brand’s goals and objectives. Employee brand engagement is beneficial for the organizations because employees who are engaged with the brand work as marketers for the firm (Yousf and Khurshid, 2021). The marketers, then, promote the organizational brand to potential employees and customers at minimal or no cost, thus increasing interpersonal interaction with stakeholders and enhancing the firm’s performance. Moreover, brand engagement is crucial for organizational performance because the willingness of the employees leads to enhanced employee performance.

Organizations develop strategies to engage the employee to induce employee brand engagement. Branding itself is a complex phenomenon that needs the attention of researchers (Venkatesh et al., 2022). Scholars have emphasized the methods taken by organizations to enhance a firm’s performance through employee brand engagement. The employees who are given the right to participate in decision-making tend to engage in employee brand engagement. Such employees perceive an organization as their own; therefore, they show high engagement toward the job and the organization (Pitt et al., 2018). Moreover, literature also showed that employee brand engagement helps the firm to achieve a competitive advantage by promoting the brand to the stakeholders. Such efforts led by the employees generate revenue and profit for the firm (Paulsen, 2016).

The literature has shown the importance of knowledge sharing that includes experiences, knowledge, and skills of the organizational members (Halisah et al., 2020). Both practitioner-related publications and academic contributions have emphasized promoting knowledge-sharing culture and disseminating information as much as possible (Azeem et al., 2021). In the process of knowledge sharing, the employees develop social interaction and interpersonal skills. Knowledge-sharing culture facilities access to knowledge and establish trust and a positive working environment for the employees (Al Kurdi et al., 2021). Knowledge is regarded as the most valuable resource for the organization. Scholars argue that sharing knowledge not only increases value for achieving competitive advantage but also develops skills among the employees.

Knowledge sharing is comprised of three significant aspects i.e., the process of knowledge sharing, the type of knowledge shared within the firm, and the approaches of knowledge sharing (Intezari et al., 2017). The two most important processes of knowledge sharing are knowledge collection and knowledge donation. Knowledge is donated by the employees who communicate their knowledge to others, while, knowledge is collected by the employees who want to gain information (Li et al., 2021). The firms that encourage the employees to share knowledge and devise favorable policies develop a knowledge-sharing culture. The equal distribution of knowledge around the organization benefits the employees and the organization in terms of high employee and organizational performance (Azeem et al., 2021).

Swift technological advancement and a competitive business environment foster the organizations to utilize their resources like human capital to enhance innovative capability (Tong et al., 2019). The innovative capability enables the organizations to develop, invent value, and attain long-term competitive advantage (Le and Le, 2019). Moreover, innovative capability helps the firms to adjust to uncertain internal and internal environments in an effective manner and leads toward the success of the organization (Hou et al., 2019). The management of the organizations is mainly responsible for promoting the innovative capability of the employees through their effective decisions and organizational policies. The innovative capabilities of the employees are also enhanced by introducing new advanced technology in the organization.

Scientific developments and technologies in the era of globalization and a fast-changing environment benefit the firms to become flexible, efficient, and responsive (Hong et al., 2019). Consequently, the employees develop innovative capabilities that benefit the organization in the long run. In addition, innovation is highly needed in this fierce business environment because without innovation the organizations cannot survive (Le and Le, 2019). Various employee-related and organizational-related factors significantly contribute to inducing innovative capabilities. For example, decision-making power, knowledge sharing culture, supportive leadership, and employee engagement are some important factors that enable the innovative capabilities of the employees (Ahmed et al., 2020).

Studies, such as (Uddin et al., 2019) and (Sakka and Ahammad, 2020), have been undertaken to investigate the role of employee engagement on different organizational factors. For example, (Uddin et al., 2019) examined the impact of employee engagement on team performance with the mediation of organizational citizenship behavior and employee commitment. In addition, (Sakka and Ahammad, 2020) analyzed the influence of employee brand ambassadorship on employee usage with the mediating role of employee wellbeing in the context of social media. The authors of these studies suggested inculcating different organizational and employee-related factors in the existing model. Therefore, the present study aims to investigate the impact of two important organizational and employee-related factors i.e., employee brand engagement and knowledge sharing culture on enterprise performance. Organizational behavior literature demonstrated the need to examine the factors that lead to enterprise performance. To the best of the author’s knowledge, a lack of evidence is present related to the mediating role of innovative capabilities in the organizational context. In addition, studies like (Sharma et al., 2021) emphasized investigating the antecedents and precedents of innovative capabilities. Thus, the study intends to analyze the mediating role of innovative capability in the relationship between employee brand engagement and enterprise performance and between knowledge sharing culture and enterprise performance.

The present study developed a few objectives based on the literature, which are to: examine the impact of employee brand engagement on enterprise performance, investigate the influence of knowledge sharing culture on enterprise performance, analyze the role of employee brand engagement on innovative capabilities, and determine the role of knowledge sharing culture on innovative capabilities. The study also established the objectives related to the mediating role of innovative capabilities, which are to: investigate the mediating role of innovative capabilities in the relationship between employee brand engagement and enterprise performance and analyze the mediating role of innovative capabilities in the relationship between knowledge sharing culture and enterprise performance. The research questions have also been developed which are: what is the impact of employee brand engagement on enterprise performance? What is the influence of knowledge-sharing culture on enterprise performance? What is the role of employee brand engagement on innovative capabilities? What is the role of knowledge-sharing culture on innovative capabilities? The study also established the research questions related to the mediating role of innovative capabilities, and the questions are: do innovative capabilities mediate the relationship between employee brand engagement and enterprise performance? And do innovative capabilities mediate the relationship between knowledge sharing culture and enterprise performance?

## Review of Literature and Hypotheses Development

The study intends to analyze the role of organizational-related and employee-related factors that affect enterprise performance. In this regard, the study examined the role of employee brand engagement and knowledge sharing culture on enterprise performance in China. The study also determined the mediating effect of innovative capabilities in the relationship between employee brand engagement and enterprise performance. The framework of the study has been based on social exchange theory (SET) and employee stewardship theory (SET).

### Social Exchange Theory

The social exchange theory (SET) developed by Homans (1958) states that the exchange process affects the social behavior of individuals. This exchange aims to enhance the benefits while reducing costs. This theory also explains that people value the benefits and risks associated with social relationships. However, if the risks are more than the benefits then the individuals abandon the relationship. SET claims that when the benefits are high, the social relationship between the individuals gets stronger. In the organizational context, this social exchange results in benefiting the entire organization because the employees are willing to work effectively and develop relevant skills in an environment that encourage and promotes positive relationships (Cropanzano and Mitchell, 2016).

Based on the conceptual framework of the study, the hypotheses proposed that a knowledge-sharing culture affects innovative capabilities and enterprise performance. In the light of the social exchange theory, the theory explicitly states that the benefits obtained by the individual during the social exchange are beneficial for the organization. Knowledge-sharing culture promotes social relationships and interactions within the organization because employees communicate with one another while sharing knowledge. Consequently, the social relationship built through knowledge sharing influences the innovative capabilities of the employees and enterprise performance.

### Employee Stewardship Theory

Employee stewardship theory (EST) developed by Donaldson and Davis (2016) explains that individuals possess intrinsic motivation to work for organizations and others in order to achieve the assigned tasks and responsibilities given to them. In order words, employees who want to work for the organization put extra effort to accomplish the goals and enhance the performance of the organization. Moreover, employee stewardship theory emphasized the fact that intrinsic motivation is necessary to boost the morale of the employees to work effectively for the organization. Under the employee stewardship theory (EST), employees essentially want to do their job effectively and become good stewards of the organization.

The framework of the study demonstrated that employee brand engagement impacts innovative capabilities and enterprise performance. This is based on employee stewardship theory which also states that employees’ intrinsic motivation allows them to work effectively for the organization. Thus, intrinsic motivation (employee brand engagement) positively develops innovation capabilities of the employees to meet the goals and objectives of the organization and enhance enterprise performance. The innovative capabilities are significant for the employees because the present era requires employees who can think innovatively and creatively for the organization.

### Relationship Between Employee Brand Engagement and Enterprise Performance

Employee brand engagement is activity related to the employees’ involvement in the organization’s brand (Van Nguyen et al., 2021). It is one of the key indicators of organizational success (Ahmed et al., 2020). This type of engagement predicts the financial performance of the organization and organizational success along with the performance of the employees. It impacts the efficiency and production level of the organization, organizational reputation, customer retention, advocacy, and culture of the organization (Al-dalahmeh et al., 2018). This signifies that employee brand engagement is a highly significant aspect that not only impacts the organization but also the external organizational environment. Employees who focus on their work and are aware of their surrounding environment justify high engagement and this ultimately leads to maximum competition with minimal confusion (Linggiallo et al., 2021). Moreover, the employees who are engaged in their work and work passionately perform higher than the employees who show low engagement. Employees with high engagement contribute to the success of the enterprise. According to Alola and Alafeshat (2021), enthusiasm, inspiration, and dedication are all related to the high engagement of the employees.

The performance of the organization or enterprise is based on articulated, well-planned, and effective employee brand engagement policies and strategies. Studies have attempted to establish different techniques for developing employee brand engagement, refining employee brand engagement, and understanding this complex phenomenon (Yuniati et al., 2021). For example, He et al. (2021) examined the factors that greatly contributed to employee engagement and the results presented that equity, values, workload, communities, control, and rewards impact employee engagement. The author also discussed that transfer of information, thoughts, and ideas influence the employee brand engagement within the organization. Ahmed et al. (2020) claimed that most studies focused not only on the aspects that impact employee brand engagement but also on organizational outcomes associated with employee brand engagement. To explain this, Schneider et al. (2018) studied the impact of workforce engagement on organizational performance and revealed that organizational performance is the descendent of workforce engagement among publicly traded companies.

The relationship between employee engagement and organizational performance among information technology (IT) employees have been found by Al-dalahmeh et al. (2018) who claimed that employee engagement is closely related to organizational performance through job satisfaction. Another recent study that showed similar results was carried out by Yuniati et al. (2021) who claimed that talent management is positively and significantly related to organizational performance *via* employee engagement. Moreover, Anwar and Abdullah (2021) conducted a study on government employees of Iraq to investigate the influence of human resource practices on organizational performance. The outcomes demonstrated that employee training and engagement had a strong influence on organizational performance while decentralization has no impact on organizational performance. Most of the studies have examined the role of employee engagement on organizational performance, however, very few studies explored the impact of employee brand engagement on organizational performance. Thus, based on the literature, this study analyzed the effect of employee brand engagement on enterprise performance, and the hypothesis has been developed as follows:

***H*****_1._** *Employee Brand Engagement has an effect on Enterprise Performance*

### Relationship Between Knowledge Sharing Culture and Enterprise Performance

In this present era of a knowledge-based economy, the role of knowledge is crucial in enhancing the overall value of the organization (Singh et al., 2021). Individuals possessing high knowledge enable the organizations to extend and achieve the performance that ultimately leads toward organizational sustainability (Le and Le, 2019). Nonetheless, organizations that do not promote a knowledge-sharing culture cannot perform well in this highly competitive business environment. Prior studies, for example, Ohemeng and Kamga (2020), claimed that entrepreneurs who share and participate in the valuable knowledge enhance entrepreneurial performance which consequently impacts organizational performance. Knowledge-sharing culture helps in adopting new technology, problem-solving, inventing something new, and developing the dynamic capabilities of a firm Singh et al. (2021). These factors significantly affect the overall organizational performance by achieving the goals and objectives of the organization.

Organizational culture dynamically influences the system of learning in which employees can exchange and share ideas or job experiences through social interactions and communication (Oyemomi et al., 2019). The cognitive abilities of the employees are affected when exposed to a new culture of knowledge sharing Suchitra et al. (2020). Consequently, these abilities allow the employees to show high performance which leads to high organizational performance. In other words, knowledge sharing culture pacifies the knowledge creation environment and regulates the behavior of the employees which is highly significant for knowledge exchange and creation (Singh et al., 2021). Thus, it is imperative for the organization to encourage the environment of utilizing these cognitive abilities during socialization at work for creating, sharing, and using the knowledge.

The knowledge-sharing ability of the employees develops the capability to attain a competitive advantage (Meher et al., 2022). Researchers argued that knowledge sharing culture encourages the capabilities of the employees and organization to develop new products, involve in change, and show a willingness to increase the overall performance of the organization (Li et al., 2020). Oyemomi et al. (2019) argued that developing a knowledge-sharing culture along with, trained employees and supportive leadership boosts organizational performance. Knowledge-sharing culture helps the employees to put forward their opinions, ideas, and thoughts for the betterment of the organization (Oyemomi et al., 2019). The culture of knowledge sharing builds interpersonal trust, intention to share knowledge, personal motivation, and reciprocal relationship which consequently lead to organizational success (Li et al., 2020). To the best of the author’s knowledge, there is still room to investigate the impact of a knowledge-sharing culture on enterprise performance in different contexts. Therefore, this study postulated that:

***H*****_2._** *Knowledge Sharing Culture has an effect on Enterprise Performance*

### Relationship Between Employee Brand Engagement and Innovative Capabilities

Employee innovative capabilities rely on employee engagement (Al-Kurdi et al., 2020). The employees who are engaged with their job and organization strive to develop skills that can benefit the organization. However, employees with less engagement show poor work performance which ultimately affects the overall organizational performance (Ben Moussa and El Arbi, 2020). Such employees do not put any effort to change their behavior for the betterment of the organization. Azeem et al. (2021) claimed that training does not affect the employees with low engagement because they do not show a willingness to work for the organization. However, highly engaged employees go beyond their regular responsibilities and develop innovative capabilities that result in better performance (Ahmed et al., 2020). Employee engagement in the organization enables the employees to work effectively for the organization by learning something new, developing innovative skills, and saving organizational resources Paillé and Valéau (2020). Hence, innovative capabilities can be achieved if the employees are engaged.

The engagement of the top management toward their organization impacts the employee engagement level (Le and Lei, 2019). In brief, employee engagement to change (here, innovative capabilities) is significant for the organizational processes and ability to innovate products that fulfills the need of the market (Paulsen, 2016). Employee engagement to develop innovative capabilities positively impacts their willingness to create innovative products. Conversely, without employee engagement, the innovation would be challenging to achieve (Zheng et al., 2022). Highly engaged employees find ways to improve the operations of the organization, therefore they develop innovative capabilities that can help in better organizational performance. Moreover, substantial studies and previous literature on the relationship between employee engagement and innovation showed that engagement is a key to competitiveness and innovation (Ben Moussa and El Arbi, 2020). Hence the innovative capability is developed among the employees if they are engaged in their job or organization to achieve competitive advantage in this business environment. Highly engaged employees perform innovatively in the organization and attract potential employees.

Employee engagement leads to innovation through the innovative capabilities of the employees, and due to these capabilities, employees do beyond their roles by collaborating with their peers, making improvements for the organization, and working for the organization to stand in the business market (Al-Kurdi et al., 2020). Employee engagement assumes an important precedent for innovation and creativity in the workplace (Knox and Marin-Cadavid, 2022). The authors claimed that the social exchange theory (SET) is the theoretical bases of engagement and innovative capability of the individuals. This theory suggests that, when employees are given value in terms of training and empowerment, they feel a sense of consideration, thus, they return the favor to the organization by engaging with the organization. High engagement leads to creative and innovative behavior as the employees perform more than their responsibilities (Kwon and Kim, 2020). On the basis of the above literature and social exchange theory, the following hypothesis has been proposed:

***H*****_3._** *Employee Brand Engagement has an effect on Innovative Capabilities*

### Relationship Between Knowledge Sharing Culture and Innovative Capabilities

One of the most crucial factors that encourage innovation in the employees is a knowledge-sharing culture (Castaneda and Cuellar, 2020). Innovative behavior of the employees is unlikely to occur if the organizations do not promote a knowledge sharing culture (Kremer et al., 2019). Gaining skills and knowledge by collaborating with colleagues and peers has been an efficient and effective way to be successful in terms of innovation (Bukht and Heeks, 2017). Knowledge sharing in the context of innovation signifies the exchange of expertise for creating and improving the services and products offered by the organization. Organizations regard knowledge sharing as a resource that enables product development capability through innovation (Arsawan et al., 2022). Hong et al. (2019) claimed on the basis of a meta-analysis that a knowledge-sharing culture is closely related to team performance. However, a lack of knowledge can hinder the innovative capabilities of the employees (Intezari et al., 2017). Organizations that encourage a knowledge-sharing culture produce new ideas and develop innovative capabilities among the employees. Additionally, Castaneda and Cuellar (2020) revealed that businesses that are highly engaged in knowledge networks produce employees with higher innovative capabilities.

There are mainly two types of knowledge i.e., tacit knowledge and explicit knowledge. Singh et al. (2021) found that tacit knowledge has a stronger association with innovative capability as opposed to explicit knowledge. The reason is tacit knowledge cannot be replicated by others. However, knowledge sharing can convert tacit knowledge into explicit, and both types enable innovation in the organization. The most fruitful route that leads to innovative behavior is informal knowledge sharing which is induced if the organizations promote a culture of knowledge sharing (Kremer et al., 2019). The knowledge-sharing culture encourages the employees to be innovative in terms of the propensity to devise innovative strategies to create novel products (Halisah et al., 2020). A recent investigation also found that knowledge sharing reduces job stress and facilities the innovative behavior of the employees (Montani and Staglianò, 2022). Few studies have investigated the impact of knowledge sharing on innovation, but limited evidence is available with regards to the knowledge sharing culture and innovative capabilities, which is the objective of the present study. Several authors have identified the importance of examining knowledge-sharing culture and innovative capabilities together. On the basis of the above discussion, the following hypothesis has been posited:

***H*****_4._** *Knowledge Sharing Culture has an effect on Innovative Capabilities*

### Mediating Role of Innovative Capabilities

Organizations are developing strategies and methods to maximize the innovative capabilities of the employees for sustaining competitive advantage (Hong et al., 2019). Innovative performance directly impacts organizational performance because developing new products attract customers (Shanker et al., 2017). Moreover, employee engagement facilitates innovative behavior because when the employees are engaged, they develop a sense of consideration and find effective ways to benefit the organization. Ismail et al. (2019) investigated the influence of employee engagement on job performance with the mediation of creativity among the employees working in Lebanon and found that employee engagement and job performance are positively related to each other and creativity fully mediated the relationship. Innovative capabilities are built in the employees who feel a deeper sense of engagement with their job or organization, which in turn benefits the whole organization (Venkatesh et al., 2022).

Both motivation and employee engagement are the biggest drivers of innovative work behavior (Knox and Marin-Cadavid, 2022). Employees develop innovative capabilities as a result of intrinsic motivation and engagement with their organization or job. As employees become innovative, they strategically develop innovative methods to solve daily tasks which in turn positively influence the overall organizational performance (Le and Le, 2019). Innovative capabilities of the employees as a result of employee engagement make the employees do their job beyond their responsibilities. This results in better organizational performance and achieving a competitive advantage in the fierce business market (Al Kurdi et al., 2021). The employees who are highly engaged in their job find ways to improve the operations of the organization, therefore they develop innovative capabilities that can help in better organizational performance. Employee engagement in the organization enables the employees to work effectively for the organization by learning something new, developing innovative skills, and saving organizational resources (Paillé and Valéau, 2020). Innovative capability helps the firms to adjust to uncertain internal and internal environments effectively and leads toward the success of the organization (Hou et al., 2019).

Innovation is regarded as an important indicator of organizational success which is the key to attaining competitive advantage (Ben Moussa and El Arbi, 2020). According to Suchitra et al. (2020), the culture of the organization matters to a great extent when it comes to innovation and organizational performance. Existing research supports that a knowledge-sharing culture and innovative capabilities are important indicators of organizational performance and competitive advantage. The scholars argued that knowledge sharing and innovative capabilities are closely associated with one another (Montani and Staglianò, 2022). When these factors are aligned together, they can bring better organizational strategies and desirable organizational performance (Singh et al., 2021). Substantial studies showed the significant role of knowledge management and knowledge sharing in the development of innovative capabilities (Kremer et al., 2019). In other words, knowledge-sharing culture promotes innovative capabilities, skills, and competencies for the betterment of the organization. Prior studies, for example, Ohemeng and Kamga (2020), claimed that entrepreneurs who share and participate in the valuable knowledge enhance entrepreneurial performance which consequently impacts organizational performance.

Although studies have been conducted to examine the relationship between employee engagements, knowledge sharing culture, enterprise performance, and innovative capabilities, limited studies focused on the mediating role of innovative capabilities in the relationship between employee brand engagement and enterprise performance and between knowledge sharing culture and enterprise performance. Therefore, this study proposed that:

***H_5_.** Innovative capabilities mediate the relationship between Employee Brand Engagement and Enterprise Performance*

***H_6_.** Innovative capabilities mediate the relationship between Knowledge Sharing Culture and Enterprise Performance*

**FIGURE 1:**
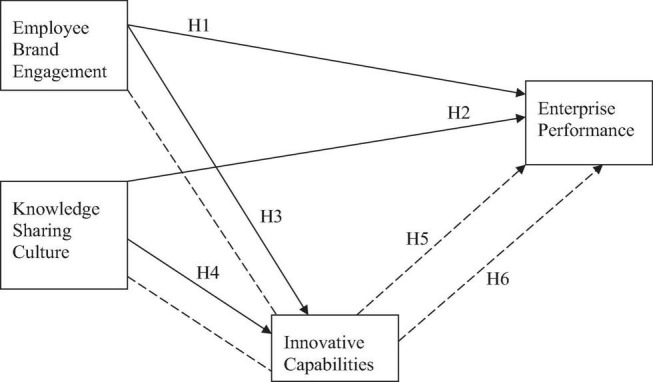
Conceptual model. EBE, employee brand equity, KSC, knowledge sharing culture, IC, innovative capabilities, EP, enterprise performance.

Figure 1 below shows the conceptual model which has been developed on the basis of the proposed hypothesis and the literature:

## Methodology

The effects of employee brand engagement and knowledge sharing culture on enterprise performance, in addition to the mediating role of innovative capabilities were checked and analyzed in this study. This research has used a quantitative research design along with deductive approach to check the above-mentioned effects and mediating relationships. In particular, the hypotheses have been developed through rigorous review of literature in the study for checking the effect of employee brand engagement and knowledge sharing culture on the enterprise performance. Quantitative research design has been used in this study to make sure that biases do not affect the analysis and results of this research. The data collection process for this research was aided through the questionnaire survey that was administered by the researcher themselves. The respondents were encouraged to stay neutral since there is no correct or false response to the questions given. The population selected in this study is the employees of information technology sector working in the software houses in China. The sampling technique used in this research was the convenience sampling as it gives the liberty to the researcher to collect data from the respondents based on the availability of the respondents and comfort of the researcher (Scholtz and Scholtz, 2021). The unit of analysis in this research was the individuals as it is collected from the employees working in the software houses in China. A total of 500 paper-based questionnaires were disseminated after ensuring the clarity of the content of the questionnaire to the participants and precision in understanding to ensure the rationality of data collected. The questionnaires received that were finally used in the analysis of data were 323 (response rate of 64.6%). The ethics of the research have been fulfilled by making the respondents easy and comfortable to freely fill the questionnaire, as their responses were kept anonymous. The responses obtained from the respondents were analyzed using the statistical software to check the hypotheses. However, before checking the hypotheses, the data was screened for the initial validation if it showed reliability and validity.

### Statistical Tool

Structural equation modeling (SEM) analysis has been employed on the data to reach the concluding behavior of the data. It has followed the partial least square approach using the software Smart PLS. This helps the researcher to analyze data with the help of path modeling in a short time (Hair et al., 2014). Through this software, the variable that act as dependent variable in one relationship behaves as the independent variable in subsequent relationship without affecting the other paths (Meng and Bari, 2019). The data is analyzed through measurement and structural model stages. These help the researcher to screen the data using validity, reliability and support or deject the hypotheses formulated based on the significance of the results.

### Scales

A five-point Liker scale-based questionnaire has been used by the researchers to collect data from the respondents. Description of the scales used for each variable has been given in below.

Scale for the independent variable of employee brand engagement consisting of nine items has been borrowed from Tsourvakas and Yfantidou (2018). The sample item includes “I think my job is considered important in my company.” The scale for the second independent variable of knowledge sharing culture consisting of three items has been borrowed from Kim and Lee (2006). The sample item includes “I can freely access documents, information, and knowledge held by other divisions within the organization.” The scale for the mediating variable of innovative capabilities consisting of three items has been borrowed from Sharma et al. (2021). The sample item includes “The enterprise uses the most efficient materials during the process of product development, design, or production.” Sale for the dependent variable of enterprise performance consisting of nine items has been borrowed from Sharma et al. (2021). The sample item includes “Our firm conforms with the requirements of inputs.” These scales have helped the researcher to collect data from the potential respondents.

### Demographic Profile

Table 1 shows the demographic characteristics of the respondents. The female participation (65.94%) has been higher in the survey than male participation (34.05%). This is possibly due to the fact that post pandemic females have shown up in the offices more than the males and they agreed to be part of the survey than the males. The age had been segmented into four categories. The highest participation has been observed among the age 31–40 years (36.22%) category. However, the lowest participation is seen among the participants of age above 50 years (12.38%). Similarly, in education as well, the highest participants had bachelor degree (45.82%) while the lowest number of participants had Ph.D degree or other certifications or diplomas which made 26.93%.

**TABLE 1:** Demographics analysis.

Demographics	Frequency	Percentage
**Gender**		
Male	110	34.05%
Female	213	65.94%
**Age (years)**		
20 – 30	114	35.29%
31 – 40	117	36.22%
41 – 50	52	16.09%
Above 50	40	12.38%
**Education**		
Bachelor	142	45.82%
Master	94	29.10%
Ph.D. and other studies	87	26.93%

*N = 323. Other studies refer to diploma, certification or affiliation.*

## Data Analysis and Results

### Measurement Model

**FIGURE 2:**
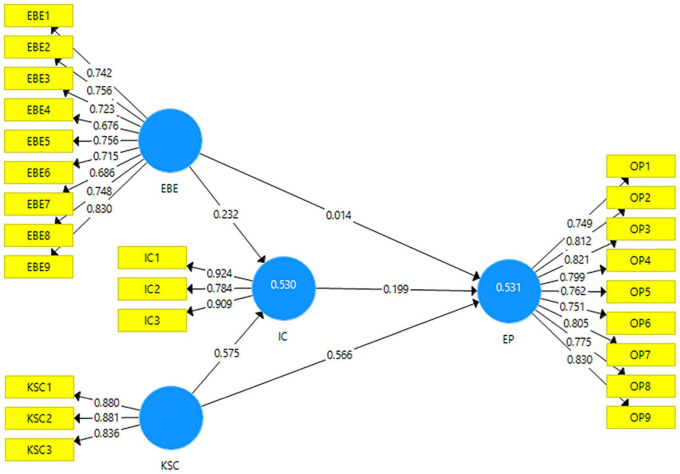
Measurement model. EBE, employee brand equity, KSC, knowledge sharing culture, IC, innovative capabilities, EP, enterprise performance.

Figure 2 explains he measurement model of the analysis. It can be seen in the following.

Results obtained through the Figure 2 are given in the Table 2. According to Bollen (2019), the factor loadings for the items of each variable are considered satisfactory if they are above 0.6. Results obtained for this research show that all the values obtained for factor loadings are above the criteria mentioned. The minimum factor loading obtained in this research is for 0.676 which is for the item EBE4. The variance inflation factor (VIF) is a collinearity diagnostic mostly used by the researchers. According to Salmerón et al. (2018), its values should be below 5.5 so to not have the issue of multicollinearity in the data. In this research, all the values are found below the maximum criteria. The maximum value of VIF obtained in this research is 4.6 hence not showing any indications for multicollinearity.

**TABLE 2:** Measurement model assessment.

				Construct reliability and validity		
				
Variables	Factor loadings		VIF	α	Composite reliability	AVE
	EBE1	0.742	1.990			
Employee brand equity	EBE2	0.756	2.720			
	EBE3	0.723	2.473			
	EBE4	0.676	1.614	0.898	0.915	0.545
	EBE5	0.756	3.765			
	EBE6	0.715	2.944			
	EBE7	0.686	2.827			
	EBE8	0.748	3.940			
	EBE9	0.830	2.889			
Innovative capabilities	IC1	0.924	3.820			
	IC2	0.784	1.467	0.925	0.937	0.624
	IC3	0.909	3.603			
	KSC1	0.880	2.131			
Knowledge sharing culture	KSC2	0.881	2.168	0.843	0.907	0.765
	KSC3	0.836	1.688			
	OP1	0.749	3.169			
	OP2	0.812	4.559			
	OP3	0.821	3.651			
	OP4	0.799	1.983			
Enterprise performance	OP5	0.762	4.357	0.833	0.900	0.750
	OP6	0.751	3.928			
	OP7	0.805	4.632			
	OP8	0.775	3.925			
	OP9	0.830	1.990			

Cronbach alpha and composite reliabilities have been used in this research to check the construct reliability. In order to show the internal consistency in the data, Taber (2018) says that the value of Cronbach alpha and composite reliability should be above 0.7. In this research, all the values for the Cronbach and composite reliability are above these criteria. The lowest value for the Cronbach alpha obtained is 0.833 for the variable enterprise performance, hence showing the internal consistency of the variables. The next indicator for the convergent validity of the data used in this research is average variance extracted (AVE). According to Shrestha (2021), value of AVE above 0.5 indicates the occurrence of convergent validity in the data. In this research, the values obtained for AVE are above this criterion. The minimum value obtained for AVE is 0.54 for the variable employee brand equity which shows that the present research meets the criteria of convergent validity.

The present research has also checked the discriminant validity of the scales using Fornell and Larcker criterion and HTMT (Heterotrait-Monotrait) ratio. Results of these tests are reported in the Table 3. According to Fornell and Larcker (1981), the scales are said to be valid if the highest value in the test is at the top of each column. It can be seen in Table 3 that 0.738 is the highest value in the column of employee brand equity. Hence, the results indicate that scales show discriminant validity. The second measure is the HTMT ratio. According to Franke and Sarstedt (2019), HTMT results are considered significant if the values for the ratio of the correlations between two variables are less than 0.85. The results of the HTMT ratio in Table 3 show that all the values are less than the described criteria, hence confirming the discriminant validity.

**TABLE 3:** Discriminant validity.

Fornell–Larcker criterion					Heterotrait–Monotrait ratio				
	
	EBE	EP	IC	KSC		EBE	EP	IC	KSC
EBE	0.738				EBE				
EP	0.433	0.790			EP	0.435			
IC	0.546	0.605	0.874		IC	0.592	0.674		
KSC	0.547	0.714	0.702	0.866	KSC	0.595	0.800	0.838	

*EBE, employee brand equity, KSC, knowledge sharing culture, IC, innovative capabilities, EP, enterprise performance.*

The coefficient of determination (r-square) explains the sustainability of the model fit indicating how much obtained values fit the regression line (Akossou and Palm, 2013). A coefficient of determination near 50% or 0.5 indicates a good model fit. In this research, the dependent variable of enterprise performance shows the r-square of 53.1% and the innovative capability shows the r-square of 53%. This shows that the present model of the research is sustainable and possesses good model fit. The f-square is also an indicator of the model fit checked through the effect size of the variables. According to Selya et al. (2012) the reference value for a small effect size is 0.02, medium effect size is 0.15 and large effect size is declared if the f-square value is 0.35 and above. In this research, large effect size (f-square value) has been seen between knowledge sharing culture and innovative capabilities which is 0.49. Another large effect size is observed between the variable knowledge sharing culture and enterprise performance which is 0.32. In addition, small effect sizes have been observed between the variable’s employee brand equity and innovative capabilities (f-square = 0.08). Another small effect size is also observed between the variable’s innovative capabilities and enterprise performance (f-square = 0.04). Furthermore, the predictive relevance of the variables has also been calculated in this research to endorse the model fit. According to Huang (2018), for predictive relevance to be significant should be above zero. In this research, the q-square values obtained are above zero indicating significant predictive relevance of the variables (enterprise performance = 0.30 and innovative capabilities = 0.38).

### Structural Model

**FIGURE 3:**
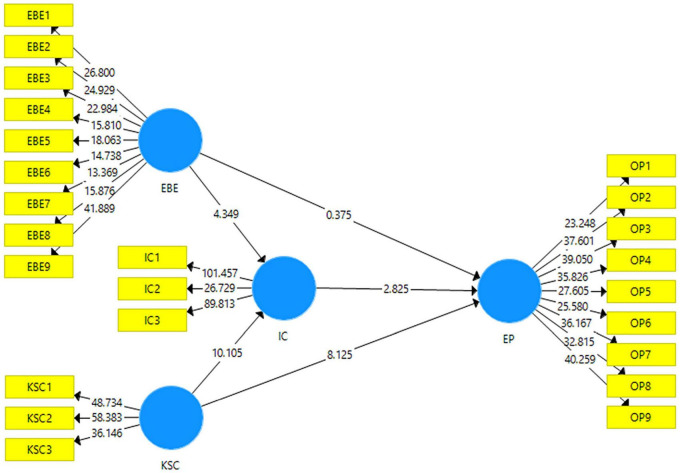
Structural model.

Figure 3 gives the pictorial elaboration of the structural model which is obtained by running the bootstrapping with 5,000 subsampling. The statistics used for the acceptance of these hypotheses are t-statistics and *p*-values. According to Winship and Zhuo (2020), the acceptance criteria of t-statistics is 1.96 while for *p*-value it should be less than 0.05.

There are total six hypotheses in this study. Four hypotheses are checking the direct effects of the independent variables on the dependent variables. Table 4 reports the results for the direct effects. The first hypothesis of the research states that employee brand engagement has an effect on enterprise performance. The t-statistics and *p*-value reported for this relationship are (*t* = 0.375; *p* = 0.708), hence rejecting H1. The second hypothesis of the research states knowledge sharing culture has an effect on enterprise performance. The t-statistics and *p*-value reported for this relationship are (*t* = 8.125; *p* = 0), hence accepting H2. The third hypothesis of the research states that employee brand engagement has an effect on innovative capabilities. The t-statistics and *p*-value reported for this relationship are (*t* = 4.349; *p* = 0), hence accepting H3. The fourth hypothesis of the research states that employee brand engagement has an effect on innovative capabilities. The t-statistics and *p*-value reported for this relationship are (*t* = 10.105; *p* = 0), hence accepting H4.

**TABLE 4:** Direct effects of the variable.

Paths	H	O	M	SD	T-statistics	*P*-value	Results
EBE - > EP	H_1_	0.014	0.016	0.038	0.375	0.708	*Rejected*
KSC - > EP	H_2_	0.566	0.572	0.070	8.125	<0.001	*Accepted*
EBE - > IC	H_3_	0.232	0.234	0.053	4.349	<0.001	*Accepted*
KSC - > IC	H_4_	0.575	0.574	0.057	10.105	<0.001	*Accepted*

*EBE, employee brand equity, KSC, knowledge sharing culture, IC, innovative capabilities, EP, enterprise performance.*

Table 5 reports the results for the indirect effects of the study. The first indirect effect of this research is about innovative capabilities mediate the relationship of employee brand engagement and enterprise performance. The t-statistic and *p*-value reported for this relationship is (*t* = 2.169; *p* = 0.031), hence accepting H5. The second indirect effect of this research is about innovative capabilities mediate the relationship of knowledge sharing culture and enterprise performance. The t-statistic and *p*-value reported for this relationship is (*t* = 2.959; *p* = 0.003), hence accepting H6.

**TABLE 5:** Indirect effects of the variable.

Paths	H	O	M	SD	t-statistics	*P*-value	Results
EBE - > IC - > EP	H_5_	0.046	0.046	0.021	2.169	0.031	*Accepted*
KSC - > IC - > EP	H_6_	0.115	0.111	0.039	2.959	0.003	*Accepted*

*EBE, employee brand equity, KSC, knowledge sharing culture, IC, innovative capabilities, EP, enterprise performance.*

## Discussion

The gap in the literature was addressed by examining the impact of employee brand engagement and knowledge sharing culture on enterprise performance with the mediation of innovative capabilities. The direct relationships between the variables were examined i.e., the effect of employee brand engagement on enterprise performance, the effect of knowledge sharing culture on enterprise performance, the effect of employee brand engagement on innovative capabilities, and knowledge sharing culture on innovative capabilities. The indirect or mediating effect of innovative capabilities was also analyzed in the present study which states that innovative capabilities mediate the relationship between employee brand engagement and enterprise performance and innovative capabilities mediate the relationship between knowledge sharing culture and enterprise performance. The study from the results obtained presented some important insights into the organizational dynamics.

The first hypothesis posited that employee brand engagement has an effect on enterprise performance. The results of this hypothesis revealed that the relationship between employee brand engagement and enterprise performance is insignificant. These findings were contrary to the findings of Anwar and Abdullah (2021) who found that employee training and engagement had a strong influence on organizational performance. The possible reason could be that employee brand engagement cannot directly contribute to organizational performance without the presence of any other employee-related or organizational-related factor. The second hypothesis got accepted which posited that a knowledge-sharing culture has an effect on enterprise performance. Meher et al. (2022) found similar results which revealed that the knowledge-sharing ability of the employees develops the capability to attain a competitive advantage. Knowledge is an important asset for the organization, thus promoting a culture of knowledge sharing not only develops the interpersonal skills of the employees but also the firm’s performance.

The third hypothesis posited that employee brand engagement has an effect on innovative capabilities. The results of this hypothesis revealed that the relationship between employee brand engagement and innovative capabilities is significant. These results are in harmony with the findings of Paillé and Valéau (2020) who revealed that employee engagement in the organization enables the employees to work effectively for the organization by learning something new, developing innovative skills, and saving organizational resources. The reason is the ability of engaged employees to work effectively for the organization by developing innovative skills and capabilities. The fourth hypothesis got accepted which posited that a knowledge-sharing culture has an effect on innovative capabilities. Similar results were found by Halisah et al. (2020) who revealed that the knowledge-sharing culture encourages the employees to be innovative in terms of the propensity to devise innovative strategies to create novel products. Innovative capabilities are built when employees receive knowledge from their peers and colleagues, thus knowledge management culture is important for developing innovative behavior.

The fifth hypothesis got accepted which posited that innovative capabilities mediate the relationship between employee brand engagement and enterprise performance. These results are synchronous with the findings obtained by Al-Kurdi et al. (2020) who stated that innovative capabilities of the employees as a result of employee engagement make the employees do their job beyond their responsibilities. This results in better organizational performance and achieving a competitive advantage in the fierce business market. The reason is innovative capabilities are developed as a result of high employee brand engagement and these factors together enhance the overall performance of the enterprise. The sixth hypothesis also got accepted which posited that innovative capabilities mediate the relationship between knowledge sharing culture and enterprise performance. These results are in synchrony with the findings of Suchitra et al. (2020) who claimed that the culture of the organization matters to a great extent when it comes to developing innovative behavior and improving organizational performance. Organizations that encourage a knowledge-sharing culture induce innovative capabilities of the employees which result in better organizational performance.

## Theoretical Implications, Practical Implication, Limitations and Future Direction, and Conclusion

### Theoretical Implications

This research contributes significantly to the theory of organizational behavior and performance management. Firstly, the role of employee brand equity in the enterprise performance and innovative capabilities has been examined. Such setting of variables has not been examined before; hence, this study has significantly contributed to the management literature. Secondly, this research has found that employee brand equity does not play any role in the enterprise performance; rather it significantly impacts the innovative capabilities of the organizations. The literature has also been enriched by examining the role of knowledge sharing culture in enterprise performance and innovative capabilities. Thirdly, it has been found that knowledge sharing culture plays significant role in improving the enterprise performance through encouraging the innovative capabilities. This research has also explored the mediating role of innovative capabilities between the knowledge sharing culture, employee brand equity and the enterprise performance. Consequently, this research is vital as it gives insight into the role of knowledge sharing culture, innovative capabilities and employee brand equity in overall enterprise’s performance.

### Practical Implications

This research gives some solid practical implications for the organizations and corporate sector based on the findings of the study. Firstly, it is important for the organizations in corporate sector and software houses in information technology sector to encourage the knowledge sharing culture to enhance their overall enterprise performance. The study has shown that when the knowledge sharing culture prevails in the organizations, performance of the enterprise’s flourishes. Secondly, when the employees owning brand equity work for organizations, it helps them nourish the innovative capabilities of the organizations through empowering the coworkers by engaging them with productive processes. The human resource department can then decide whether the branded employee should be paid extra for this extra role behavior and to retain them. Similarly, there lies a responsibility with the organizations and the human resource department to avail the maximum skills of the employee brand equity by creating the opportunities for the other employees through training sessions where the elite employees share their experiences. Furthermore, this study will help the top management of the organizations to invest in the employee brand equity by hiring them for the betterment of the organizational overall environment because it supports in the enhancement of the innovative capabilities which ultimately contribute to the performance of the enterprise.

### Limitations and Recommendations

This research has been carried out in the information technology sector, considering the role of employee brand equity and knowledge sharing culture in the enterprise performance with mediation of innovative capabilities. The current study has used the sample of employees working in the software houses which limits the findings of the study. This research is based on the conceptual understanding of the researcher which opens new avenues for exploring other dimensions in different dynamics therefore, it can be checked in other working setups like construction industry, manufacturing industry or assembling industry can also be considered to get more insight into the conceptual model. This study can yield more interesting results if conducted in the European setting where wages and the labor laws are different from China that how employee brand equity plays its part in enterprise performance and innovative capabilities. Therefore, it is encouraged to explore more aspects to discuss the framework and findings of this research that will enlighten and validate the aforementioned relationships. Hence, empirical testing of the proposed framework is encouraged by introducing other important mediating factors like emotional intelligence, employee absorptive capacity or moral disengagement or the moderating variables like gender, employee well being. It will also be interesting to check the impact of employee brand equity and knowledge sharing culture in producing the employee ambassadorship in organizational setting.

## Conclusion

Enterprise performance is the most critical element for the organizations. Therefore, organizations keep on finding new ways and devise means to enhance their performance. In this study, certain determinants have been found that are important for the organizations in enhancing their performance. In present study, the role of employee brand equity and knowledge sharing culture has been examined in enterprise performance. Further, the mediating role of innovative capability has also been checked. Results of this research have described that employee brand equity does not play a role in the enterprise performance; however, knowledge sharing culture is an important determinant of the enterprise performance. The structural equation modeling also revealed that innovative capability significantly mediates the relationship of employee brand equity with enterprise performance; and the relationship of knowledge sharing culture and enterprise performance. It also offers a significant contribution to the literature by testing a comprehensive model on the employee brand equity and enterprise performance in the organizational setting in China. There are some implications as well for the human resource departments of the organizations in catering the branded employees by offering extra compensations for their extra role behaviors they exhibit in the organization in the form of innovative capabilities.

## Data Availability Statement

The original contributions presented in the study are included in the article/supplementary material, further inquiries can be directed to the corresponding author.

## Author Contributions

The author confirms being the sole contributor of this work and has approved it for publication.

## Conflict of Interest

The author declares that the research was conducted in the absence of any commercial or financial relationships that could be construed as a potential conflict of interest.

## Publisher’s Note

All claims expressed in this article are solely those of the authors and do not necessarily represent those of their affiliated organizations, or those of the publisher, the editors and the reviewers. Any product that may be evaluated in this article, or claim that may be made by its manufacturer, is not guaranteed or endorsed by the publisher.
